# Using sperm morphometry and multivariate analysis to differentiate species of gray *Mazama*

**DOI:** 10.1098/rsos.160345

**Published:** 2016-11-09

**Authors:** Marina Suzuki Cursino, José Maurício Barbanti Duarte

**Affiliations:** 1 Deer Research and Conservation Center (NUPECCE, Núcleo de Pesquisa e Conservação de Cervídeos), Department of Animal Science, Via de Acesso Professor Paulo Donato Castellane s/n, Jaboticabal, SP, 14884-900, Brazil; 2 Graduate Program in Veterinary Medicine, Doctorate in Animal Reproduction Science, Faculty of Agricultural and Veterinary Sciences (FCAV)-São Paulo State University (UNESP), Via de Acesso Professor Paulo Donato Castellane s/n, Jaboticabal, SP, 14884-900, Brazil

**Keywords:** spermatozoon, morphometry, taxonomy

## Abstract

There is genetic evidence that the two species of Brazilian gray *Mazama*, *Mazama gouazoubira* and *Mazama nemorivaga*, belong to different genera. This study identified significant differences that separated them into distinct groups, based on characteristics of the spermatozoa and ejaculate of both species. The characteristics that most clearly differentiated between the species were ejaculate colour, white for *M. gouazoubira* and reddish for *M. nemorivaga*, and sperm head dimensions. Multivariate analysis of sperm head dimension and format data accurately discriminated three groups for species with total percentage of misclassified of 0.71. The individual analysis, by animal, and the multivariate analysis have also discriminated correctly all five animals (total percentage of misclassified of 13.95%), and the canonical plot has shown three different clusters: Cluster 1, including individuals of *M. nemorivaga*; Cluster 2, including two individuals of *M. gouazoubira*; and Cluster 3, including a single individual of *M. gouazoubira*. The results obtained in this work corroborate the hypothesis of the formation of new genera and species for gray *Mazama*. Moreover, the easily applied method described herein can be used as an auxiliary tool to identify sibling species of other taxonomic groups.

## 1. Introduction

The family Cervidae is recognized as one of the most controversial due to its intergeneric relationships and the systematic taxonomy of the species within the Cetartiodactyla order. The genus *Mazama*, is known to be the most complex [1,2] and is separated into two clades, the gray (*M. gouazoubira* and *M. nemorivaga*) and the red *Mazama* (*M. americana, M. bororo* and *M. nana*) [3]. In the red clade, the species *M. americana* has recently been considered a complex of cryptic species, because different cytotypes exist in different regions of Brazil [4] and different degrees of reproductive isolation exist between these cytotypes [5]. Regarding the gray clade, phylogenetic analyses based on mtDNA, cytochrome *b* (Cyt*b*) and cytochrome *c* oxidase subunit II (COX II) have shown significant genetic differences, not only between red and gray *Mazama*, but also between *M. gouazoubira* and *M. nemorivaga*, and even among individuals within the species studied [3,6,7]. The phylogenetic distances verified led the authors to discuss the creation of new genera for each species of gray *Mazama* [3], including the possible existence of subspecies or cryptic species within each of the new taxonomic groups [6]. It is hypothesized that both species had separated into different clades around 4–5 million years ago, before their entry into South America during the Pliocene (2.5–3 million years ago) [3]. In South America, their population had distributed in different ecosystems. Nowadays, *M. nemorivaga* occupies the region of the Amazon River basin, while *M. gouazoubira* are distributed all over Brazilian territory, except the Amazonian rainforest [8]. However, there is no information on whether the species were sympatric, parapatric or allopatric before they came to South America.

Differences between sperm traits are widely used to understand divergences between species that present a strong possibility of being due to sexual selection pressure [9–12]. The evolution of different characteristics in mammalian spermatozoa probably occurred due to two main selection forces, the selection of the female reproductive tract and sperm competition [13]. There is increasing evidence that sexual selection acts on species divergence through the development of prezygotic isolation [14]. In eutherian mammals, the female selection, represented by differences in the reproductive biology of species, can direct the evolution of different sperm sizes, period of gamete fertility, amount of sperm produced or even reproductive characteristics, as genitalia morphology [15–17]. In a study on sea urchins, a single population diverged into two species that differentiated only in the locus binding sequence present in mtDNA; this gene is responsible for gamete recognition and, consequently, can lead to changes in sperm morphology [18]. The variation in sperm morphometry played a crucial role in the reproductive isolation of populations of *Ctenomys*, which together with the karyotype variation, led to speciation of the genus [19]. However, differences on morphometry related to sperm competition seem to have most important role at intraspecific competition than interspecific. Indeed, intravariation of sperm size and number have shown significant differences for males within same species [20,21]. The individual variation in sperm size may vary strategically with sperm haploid gene expression, because sperm morphogenesis occurs during the final postmeiotic phase [22,23].

Previous studies have shown significant differences in sperm head size between the two species of gray *Mazama* [24]. *Ex situ* observations of reproductive behaviour between *M. gouazoubira* and *M. nemorivaga* have indicated a fragile prezygotic barrier. (J Carranza, M Roldána, FC Peroni Ede, JMB Duarte 2016, unpublished data. Weak premating isolation between two parapatric brocket deer species.) Additionally, interbreeding between the two species has resulted in individuals with varying degrees of infertility, suggesting a strong postzygotic barrier [25]. In this study, using a multivariate analysis to distinguish different species by sperm morphometry [26] and a comparison of semen characteristics between *M. gouazoubira* and *M. nemorivaga*, we expect to strengthen the discussion on the taxonomic classification of species of gray *Mazama*, and determine the main differences in the reproductive characteristics of males between the species.

## 2. Material and methods

### 2.1 Animals

Five males were used, three adult males of *M. gouazoubira* from São Paulo State (G1, 19.7 kg body weight (22.7343° S, 47.6481° W); G2, 25.0 kg body weight (22.9099° S, 47.0626° W); and G3, 17.5 kg body weight (21.3602° S, 48.2283° W)), and two adult males of *M. nemorivaga*, one from Mato Grosso State (N1, 15.85 kg body weight (11.4229° S, 58.7572° W)) and other from Maranhão state (N2, 15.0 kg body weight (5.5206° S, 47.4718° W)). All bucks were maintained in 12 m^2^ masonry bays inside the gray *Mazama* management building at Deer Research and Conservation Centre (*Núcleo de Pesquisa e Conservação de Cervídeos*, NUPECCE). All animals were provided the same diet consisting of 500 g per deer per day of high palatability, industrialized horse feed (Purina^®^; Omolene tradicional), and 1 kg per deer per day of forage: soya bean (*Glycine max*), perennial soya bean (*Neonotonia wightii*), ramie (*Boehmeria nivea*) and mulberry (*Morus alba*). Water was provided ad libitum, and the individuals were exposed to natural fluctuations of light, temperature and relative humidity. The study was assessed by the Ethics Committee on Animal Use (CEUA) of the Faculty of Agricultural and Veterinary Sciences of São Paulo State University, which certified its compliance with the ethical principles for animal experimentation adopted by the Brazilian College of Animal Experimentation (COBEA), under protocol no. 014080/12.

### 2.2. Semen collection

Gray *Mazama* do not have reproductive seasonality, they are capable to reproduce throughout the year [27]. Ten semen collections, two per deer, were performed by electroejaculation, at different times over the period of a year. For bucks with a reproductive rest of more than one month, semen was collected 7 days before the study and discarded. For the electroejaculation procedure, the methodology described by Favoretto [28] was performed. Deer were anaesthetized intramuscularly with a combination of anaesthetics, xylazine (1 mg kg^−1^) and ketamine hydrochloride (7 mg kg^−1^). Before introducing the electrode, the rectum was emptied so that minimal interference would occur due to the presence of faeces. The eletroejaculator (P-T Electronics^®^; Boring, OR, USA) was coupled to a probe that was 2.0 cm in diameter and 28.0 cm in length, with three longitudinal electrodes along its surface, 1.5 cm apart. Following sedation and the introduction of the electrodes ventral to the rectum, each buck was submitted to electroshocks increasing from 250 mA to 750 mA, with a mean duration of 3 s, and a 3 s delay between each shock (10 sequential shocks). Three stimulation sequences were performed at intervals of 1–2 min during collection [29]. The semen was collected in 2 ml microtubes and maintained in a water bath at 36–37°C until the procedures that followed. Semen volume was determined using an automatic micropipette. The characteristics of the ejaculate were classified according to the observation of a single researcher, to avoid differences in determining sperm colour. An aliquot of 10 µl of semen was fixed in 2 ml of 10% formalin solution to determine sperm count by spermatozoon counts in a Neubauer haemocytometer chamber.

### 2.3. Sperm morphometry

Smears were performed with 5 µl of fresh semen. The slides were dried at room temperature and fixed in 3% glutaraldehyde and 4% paraformaldehyde in 0.06 M phosphate buffer for 24 h. After fixing, the slides were stained with Harris–Shorr stain [30] and observed under an Olympus Bx60^®^ light microscope (Olympus Optical do Brasil Ltda, São Paulo, Brazil). The images were captured on a Zeiss^®^ AxioCam MRm camera (Carl Zeiss MicroImaging GmbH, Göttingen, Germany).

**Figure 1.**
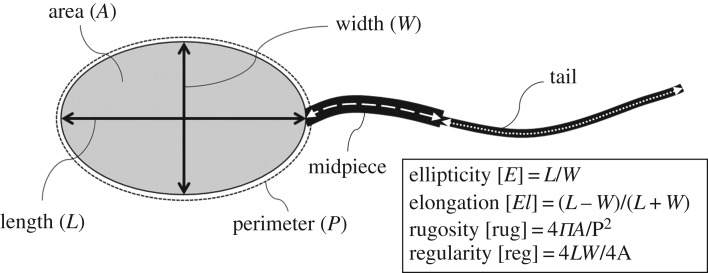
Measurements of the sperm head performed using the AxioVision v.4.8.2^®^ software: length (*L*), width (*W*), area (*A*), perimeter (*P*), midpiece and tail. The following spermatozoon characteristics were calculated based on these measurements: elongation, ellipticity, rugosity and regularity.

The measurements were made using the AxioVision 4.8.2^®^ software (Carl Zeiss MicroImaging GmbH, Göttingen, Germany). The heads of 600 spermatozoa per individual were measured, 300 spermatozoa per collection. The lengths of the tail (principal and end pieces) and the midpiece were measured in 200 spermatozoa per individual, 100 spermatozoa per collection. The dimensions of the head (area, length, width, perimeter, elongation, ellipticity, rugosity and regularity) and flagellum (tail and midpiece) were measured, as described in figure 1. Three repetitions of the measurements of each spermatozoon were performed and the means were used for statistical analyses.

### 2.4. Data analysis

Previous morphological analyses cite high values of pathologies, principally in relation to the tail (both species), abnormal head shapes for *M. gouazoubira* and middle piece defects for *M. nemorivaga* [31]. To prevent these defects from interfering in the calculations, a blot spot analysis was performed for all variables and the outliers were excluded from analyses.

#### 2.4.1. Univariate analysis

The variables volume, sperm count, head, midpiece, tail and total length were analysed and presented as the mean and standard deviation by animal (G1, G2, G3, N1 and N2). Normal distribution was tested for all variables and Tukey's test was performed for volume, sperm count, head, tail and total length. The variable midpiece had not shown normal distribution, thus a Kruskal–Wallis test was performed for comparison. The proportions of sperm regions: head, midpiece and tail were analysed by species.

#### 2.4.2. Multivariate Analysis

From the 600 spermatozoa measured, 200 spermatozoa were randomly chosen for multivariate analysis. Normality tests were performed for head dimensions variables (area, length, width, perimeter, elongation, ellipticity, rugosity and regularity) grouped by animal (G1, G2, G3, N1 and N2) and species (*M. gouazoubira* and *M. nemorivaga*). Logarithmic transformation was used for length, width, regularity and rugosity variables. It was not possible to normalize the data distribution for species; only after separating the animal G3 from *M. gouazoubira* did the data present a normal distribution. Therefore, the species were separated into three groups: *M. gouazoubira* 1, including animals G1 and G2; *M. gouazoubira* 2, including the animal G3; and *M. nemorivaga*, including animals N1 and N2. The Box's M test of equality of covariance matrices was performed for variables grouped by animals and species. As a result for both tests, the covariance matrices of the dependent variables were not equal across groups. Thus, the quadratic discriminant method was chosen for multivariate analyses. Two discriminant analyses were performed: first with three groups (species), and the second with five groups (animals). A stepwise test was performed to determine the most significant variables to perform each discriminant analysis.

The complete data from statistical analyses can be accessed at Dryad Digital Repository (http://dx.doi.org/10.5061/dryad.6471f).

## 3. Results

**Figure 2.**
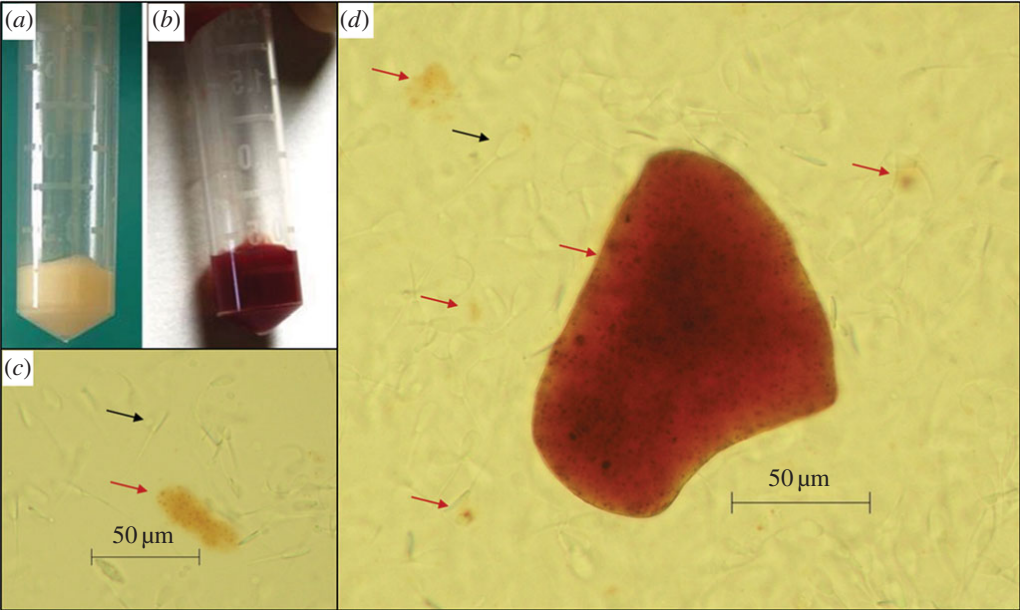
(*a*) *Mazama gouazoubira* ejaculate collected by electroejaculation was white in colour, with a creamy appearance; (*b*) *M. nemorivaga* ejaculate collected by electroejaculation was reddish in colour, with a watery appearance; (*c*,*d*) fresh ejaculate of *M. nemorivaga*, without staining, under a light microscope. Note the translucent sperm (black arrows) and reddish pigment clusters (red arrows). Scale bar, 50 mm.

Intriguingly, the colour of the ejaculate was markedly different between the two species. Ejaculate produced by *M. gouazoubira* was white, while for *M. nemorivaga* it was reddish. Analysis of fresh semen under a light microscope, without cell staining, revealed the presence of reddish clusters in the seminal plasma of *M. nemorivaga* (figure 2).

The comparison of means of volumes has shown no significant differences among animals (*p* > 0.05). Although the sperm count, as well the tail, head, total length and midpiece, has shown significant differences among animals (table 1). From the percentages of spermatozoon regions (head, midpiece and tail), the composition of *M. gouazoubira* 1 spermatozoa was identified as 73.03% tail, 15.36% midpiece and 11.61% head; for *M. nemorivaga* spermatozoa the composition was identified as 70.12% tail, 16.62% midpiece and 13.26% head; and for *M. gouazoubira* 2 as 73.20% tail, 16.42% midpiece and 10.38% head (table 2).

**Table 1.** Means and standard deviation of ejaculate characteristics (volume and sperm count) and sperm morphometry variables (head, tail, total length and midpiece) from individuals *of M. gouazoubira* (G1, G2 and G3), and *M. nemorivaga* (N1 and N2). Letters from ‘a’ to ‘q’ represent the different groups revealed by Tukey's test. * indicates variable analysed by Kruskal–Wallis test. ** indicates significance with *p*-value < 0.05.

males	*N*	volume mean ± s.d.	sperm count mean ± s.d.	head mean ± s.d.	tail mean ± s.d.	total length mean ± s.d.	midpiece* mean ± s.d.
G1	117	385.00 ± 134.57^a^	501.79 × 10^6^ ± 173.78^c,d^	8.53 ± 0.4642^g^	53.04 ± 1.39^j^	63.71 ± 1.567^o^	10.67 ± 0.477**
G2	117	638.33 ± 343.99^a^	649.59 × 10^6^ ± 328.45^c^	8.32 ± 0.3726^h^	52.96 ± 1.326^j^	64.63 ± 1.586^n^	11.63 ± 0.573**
G3	117	345.00 ± 64.03^a^	1126.7 × 10^6^ ± 256.5^b^	7.13 ± 0.3367^i^	50.24 ± 1.806^l^	61.52 ± 1.933^p^	11.27 ± 0.453**
N1	117	517.50 ± 204.13^a^	206.27 × 10^6^ ± 129.29^d,e^	9.47 ± 0.3253^f^	48.96 ± 1.534^m^	60.41 ± 1.761^q^	11.46 ± 0.512**
N2	117	318.33 ± 131.63^a^	82.775 × 10^6^ ± 60.957^e^	9.41 ± 0.3589^f^	50.91 ± 1.292^k^	63.13 ± 1.467^o^	12.21 ± 0.615**

**Table 2.** Means, standard deviation and proportions of sperm regions (head, midpiece and tail) by the total sum of spermatozoa length.

	head		midpiece		tail		
species	mean (±s.d.)	%	mean (±s.d.)	%	mean (±s.d.)	%	total (100%)
*M. gouazoubira 1*	8.42 (±0.43)	11.61	11.14 (±0.71)	15.36	53.0 (±1.36)	73.03	72.56
*M. gouazoubira 2*	7.13 (±0.34)	10.38	11.27 (±0.45)	16.42	50.24 (±1.81)	73.20	68.64
*M. nemorivaga*	9.44 (±0.34)	13.26	11.83 (±0.68)	16.62	49.93 (±1.72)	70.12	71.20

**Figure 3.**
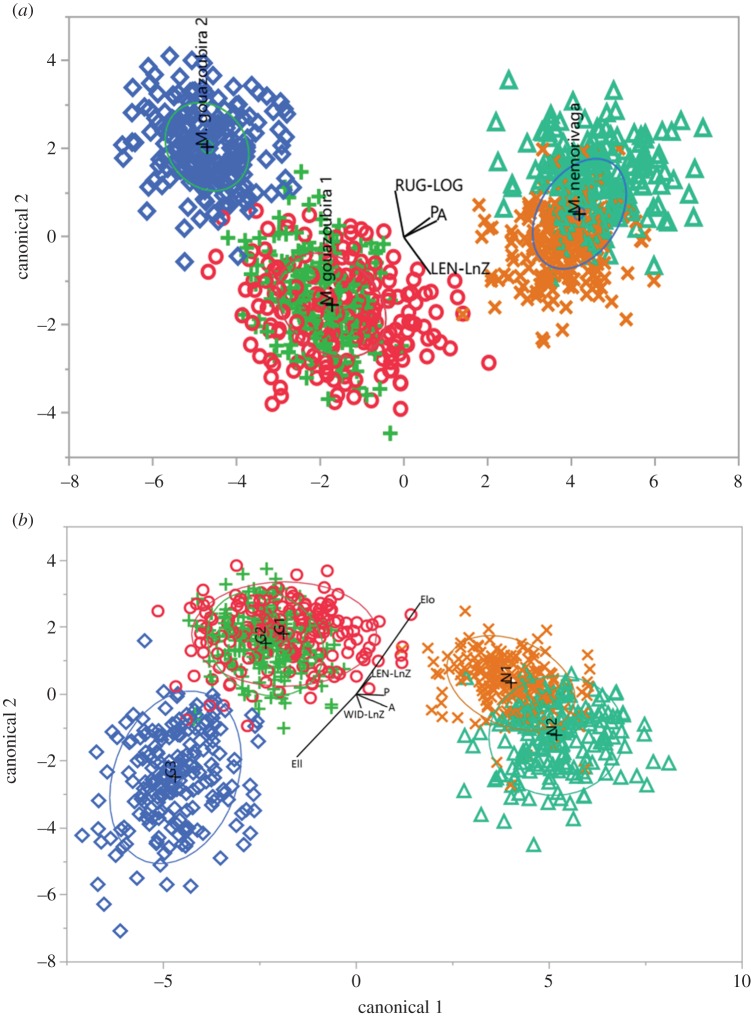
Canonical plot. The biplot axes are the first two canonical variables, Canonical 1 (*x*-axis) and Canonical 2 (*y*-axis), from the multivariable discriminant analyses. (*a*) Canonical plot of discriminant analysis by species: *M. gouazoubira* 1 (G1 (circle symbols), G2 (plus symbols)), *M. gouazoubira* 2 (G3 (diamond symbols)) and *M. nemorivaga* (N1 (cross symbols) and N2 (triangle symbols)); the rays represent the covariates used (logarithmic of rugosity (RUG-LOG), area (*A*), perimeter (*P*) and logarithmic of length (LEN-LnZ)) and their direction. (*b*) Canonical plot of discriminant analysis by animals G1 (circle symbols), G2 (plus symbols) and G3 (diamond symbols) of *M. gouazoubira* and individuals N1 (cross symbols) and N2 (triangle symbols) of *M. nemorivaga*; the rays represent the covariates used (elongation (Elo), ellipticity (Ell), area (*A*), perimeter (*P*), natural logarithmic of length (LEN-LnZ) and natural logarithmic of width (WID-LnZ)). For both canonical plots, the ellipses show the 95% confidence level for each group mean and the direction of a ray indicates the degree of association of that covariate with canonical 1 and 2. The plots were created by JMP^®^ v. 12.2.0 statistic software.

Discriminant analysis of species has separated the three groups at low error rate of identification (*M. gouazoubira* 1= 0.0125; *M. gouazoubira* 2 = 0.005; and *M. nemorivaga *= 0.0026) and total percentage of misclassified (0.71%). Discriminant analysis of animals identified the five groups with a total percentage of misclassified of 13.95% and error rate of 0.139. The error rate by animal was: N1= 0.125; N2 = 0.145; G1 = 0.195; G2 = 0.215; and G3 = 0.01. Both canonical plots, of discriminant analysis of species and discriminant analysis of animals (figure 3), have revealed the formation of three clusters in which the ellipse of 95% confidence level reveals significant difference between the clusters: Cluster 1 containing the data of *M. nemorivaga* (individuals N1 and N2); Cluster 2 containing data of *M. gouazoubira* 1 (individuals G1 and G2); and Cluster 3 containing data of *M. gouazoubira* 2 (individual G3).

## 4. Discussion

The intriguing difference in ejaculate colour between the two species occurred even though the deer receiving the same diet and were maintained under the same conditions of captivity. When observed under a light microscope, the red pigment presented in the seminal plasma forms ‘clusters’ of different sizes and formats that are not bound to the spermatozoa. Variations in colour are common in other species, ranging in tones from yellow to orange, are not indicative pathologies and are often related to the presence of riboflavin in high concentrations, which has no influence on sperm fertility [32–34]. This reddish coloration was reported by Peroni *et al.* [35], and the authors found no blood cells or haemoglobin in the seminal plasma samples of *M. nemorivaga* analysed. No other reports of seminal plasma with reddish coloration that are not related to haematospermia have been published for any other species [36,37]. Further studies on the biochemistry of the seminal plasma of *M. nemorivaga* are required to determine the chemical composition and function of this pigment in the reproductive biology of the species. This difference, the reddish clusters observed in the seminal plasma, lead us to hypothesize that the female reproductive tract environment is different between species of gray *Mazama*, although, at the moment, no study has compared semen quality or viability between those species. It seems reasonable to assume that sexual selection, affected by differences in the female reproductive tract [11,13,15], may have influenced this differentiation in seminal characteristics between the species analysed in this study. Seminal plasma plays an important role in sperm viability and transportation in the female reproductive tract [38,39], therefore, changes in the composition of the seminal plasma of different species probably co-evolved in response to sexual selection due to sperm competition [40–42].

Despite the sperm volume showing no significant differences between animals, the *M. gouazoubira* ejaculate, from G2 and G3 animals, had shown a significantly higher sperm count compared with M. *nemorivaga,* N1 and N2. It is known that the ejaculate obtained by electroejaculation is different from that obtained using an artificial vagina, because the stimulation of the electrode on the glands increases the secretion of seminal plasma and thereby decreases the ejaculate concentration, or sperm count as we named herein [43]. Moreover, it is likely that the glands of *M. nemorivaga* present greater sensitivity, given the reports that the species presents involuntary ejaculation during semen collection procedures, both in the absence of stimuli, during anaesthesia with dissociative anaesthetics, and in the presence of light stimuli, when cleaning the rectum prior to introducing the eletroejaculator [24]. Besides, the differences between the glands and the testicular size of each species should be regarded. Considering that both species are small size deer, even so, *M. nemorivaga* (approx. 15 kg) has a lower body mass compared with *M. gouazoubira* (approx. 20 kg) [44], and its sexual glands are relatively large, particularly the seminal vesicle, while the testes are relatively smaller (MS Cursino 2013, personal communication; unpublished data). These anatomical features could also explain the difference verified in sperm count between the two species. Given the larger glands, the response to the electrode stimulus is likely to be greater in *M. nemorivaga*, so the ejaculate presents an extra volume of plasma and fewer spermatozoa, which lead to a lower sperm count [45].

*Mazama gouazoubira* spermatozoa showed a head length close to the mean of the known range for Artiodactyla, which is 4.8 µm (*Tragulus javanicus ravus*) to 10.43 µm (*Philantomba monticola*) [46], whereas *M. nemorivaga* spermatozoa resemble species with larger heads. The proportions verified for the tail and midpiece were different from those reported for other deer: head length, 11–14%; midpiece, 18–21%; and tail, 66–69%, of the overall spermatozoon length [47]. The species analysed herein presented a shorter midpiece (15–16%) and a longer tail (70–73%). The main determinants of spermatozoon velocity of movement are head shape and proportions between the flagellum components, such that spermatozoa with a shorter midpiece and longer tail tend to be faster [48,49]. There is evidence that sperm competition leads to the selection of longer, faster spermatozoa [10,50,51], but the consequences of any increase in size varies among species, depending on the environment in which the sperm have to compete [52,53]. Also, the intraspecific variation on sperm morphometry found between animals, regarding head, midpiece and tail length, could be explained by the intraspecific sperm competition that occurs in most mammal species [20,21].

Although Artiodactyla shows no association between spermatozoon length and the level of sperm competition [54], our results suggest a difference in these levels between animals and species. While the two species tested possess large spermatozoa with long tails, features known to occur in fast sperm [50], *M. gouazoubira* 1 and 2 have a longer tail and smaller head compared with *M. nemorivaga*. Moreover, the species *M. gouazoubira* (including G3) shows a group of important features that indicate a more competitive environment, with greater relative testicular mass [42], a proportionally longer sperm tail in relation to head length [49], significant intraspecific difference in sperm morphometry [20,21] and, importantly, the species lives in open habitats like the Caatinga and Cerrado. Despite there being no published data regarding the Brazilian gray *Mazama* distribution or sex rate, it is likely that populations from open areas are able to meet other individuals of the same species easier and thus probably involves greater sexual competition. By contrast, the species *M. nemorivaga* has a lower relative testicular mass, its spermatozoon has a larger head and a proportionally shorter tail, and it lives in the Amazon rainforest, a closed environment that is difficult to access and where it is probably much harder to find individuals of the same species, which probably leads to decreased competition for females. However, further study on the reproductive behaviour of these species is required so a more concrete hypothesis can be developed concerning sperm competition within species of gray *Mazama*.

The spermatozoa were classified within their species with low error rate and significant differences between the clusters indicating that the variables used for the multivariate analyses were sufficiently accurate to differentiate *M. gouazoubira* 1*, M. gouazoubira* 2 and *M. nemorivaga*. Further research that provided data from other species of *Mazama* and close genera, such as *Ozotocerus*, *Hippocamelus*, *Blastocerus* and *Pudu* [3], would be interesting, in order to observe the groupings and distances formed between the different genera and species. In a similar study on the sperm morphometry of *Bos taurus* and *B. indicus*, analysis verified a higher misidentification error rate than that reported here. Despite this, it was still possible to define species-specific sperm characteristics for these bovine [55]. In the second discriminant analysis, which discriminated spermatozoa from five groups (individual identification) the classification error rate was higher among individuals within the same species than between individuals of different species.

The accuracy in identifying the spermatozoa of one *M. gouazoubira* male is worth highlighting; because the error rate was so low, it barely overlapped the classification of the two other individuals of the same species. The formation of three distinct groups of species by both discriminant analyses can corroborate with phylogenetic studies that have raised the hypothesis that *M. gouazoubira* could have distinct evolutionarily significant unit (ESU) or sibling species [6]. The accuracy achieved while identifying the spermatozoa of species of gray *Mazama* clearly indicates that further analysis, including other species of South America closely related to gray *Mazama*, also others species of genus *Mazama* and additional individuals of *M. gouazoubira* and *M. nemorivaga*, are required.

Future research, combined with recent phylogenetic studies of these species, will increase the available data and should corroborate the hypothesis of the creation of new genera of Brazilian gray *Mazama* and could lead to the identification of distinct ESUs of *M. gouazoubira*.

## 5. Conclusion

Significant differences were verified in the comparisons proposed in ejaculate characteristics and sperm morphometry, which enabled us to clearly distinguish between the species *M. gouazoubira* and *M. nemorivaga.* The accuracy achieved in discriminant analyses of sperm morphometry indicates that this should be considered a valid tool for new phylogenetic studies of the genus *Mazama*.

## Data accessibility

The complete data from statistical analyses can be accessed at Dryad Digital Repository (http://datadryad.org/review?doi=doi:10.5061/dryad.6471f) [56].
